# Caregiver burden in children with medical complexity: an approach based on the identification of associated factors

**DOI:** 10.1590/1984-0462/2026/44/2025260

**Published:** 2026-06-22

**Authors:** Fernando Sarin da Mota e Albuquerque, Danton Matheus de Souza, Lidice Valeriana Oliveira Diop, Ana Paula Scoleze Ferrer

**Affiliations:** a Universidade de São Paulo, Faculdade de Medicina, São Paulo, SP, Brazil.; b Universidade Federal de São Paulo, Escola Paulista de Enfermagem, São Paulo, SP, Brazil.

**Keywords:** Caregiver burden, Quality of life, Chronic disease, Cost of illness, Caregivers, Sobrecarga do cuidador, Qualidade de vida, Doença crônica, Efeitos psicossociais da doença, Cuidadores

## Abstract

**Objective:**

The aim of this study was to identify factors associated with increased caregiver burden of children with medical complexity and to guide management strategies.

**Methods:**

This cross-sectional study was conducted in a specialized service for patients with complex chronic conditions. Caregiver burden was assessed using the Zarit Burden Interview, which measures subjective burden, encompassing emotional, social, and personal impacts associated with caregiving, and is categorized as mild, moderate, or severe according to the total score. Bivariate analyses used chi-square tests, followed by multivariable Poisson regression with robust variance estimation, with results expressed as prevalence ratios (PRs) and 95% confidence intervals; p<0.05 was considered significant.

**Results:**

A total of 173 caregivers were interviewed, predominantly mothers (88.4%), with a mean age of 38.8 years, from low-income households. Among the patients, 28.7% had cerebral palsy, 59.4% used assistive devices, and 57.2% required help with daily life activities. A severe burden score was reported by 31.2%. Polypharmacy was associated with a higher prevalence of moderateto-severe caregiver burden (PR=1.48; p=0.015), whereas daily care support (PR=0.79; p=0.039) and adequate sleep (PR=0.78; p=0.027) were associated with a lower prevalence of the outcome.

**Conclusions:**

Care-related needs, particularly polypharmacy, daily care support, and sleep quality, were associated with caregiver burden level. These findings highlight potentially modifiable factors and support the integration of a family-centered approach in the clinical follow-up of children and adolescents with medical complexity.

## RESUMO

**Objetivo:**

Identificar fatores associados à sobrecarga do cuidador de crianças com complexidade médica e orientar estratégias de manejo.

**Métodos:**

Estudo transversal conduzido em serviço especializado em pacientes com condições crônicas complexas. A sobrecarga foi avaliada com a Escala de Sobrecarga de Zarit, que mensura a sobrecarga subjetiva, abrangendo impactos emocionais, sociais e pessoais associados ao cuidado, sendo categorizada como leve, moderada ou grave conforme a pontuação total. Foram realizados teste qui-quadrado e regressão de Poisson multivariada com estimativa robusta da variância, com resultados expressos como razões de prevalência (RP) e intervalos de confiança (IC) de 95%; p<0,05 foi considerado significativo.

**Resultados:**

Foram entrevistados 173 cuidadores, predominantemente mães (88,4%), com idade média de 38,8 anos e baixa renda. Dos pacientes, 28,7% apresentavam paralisia cerebral, 59,4% utilizavam dispositivos assistivos e 57,2% necessitavam de ajuda para as atividades da vida diária. Sobrecarga grave foi relatada por 31,2% dos cuidadores. Polifarmácia esteve associada a maior prevalência de sobrecarga moderada a grave (RP=1,48; p=0,015), enquanto o apoio no cuidado diário (RP=0,79; p=0,039) e o sono adequado (RP=0,78; p=0,027) estiveram associados a menor prevalência do desfecho.

**Conclusões:**

Características relacionadas ao cuidado, particularmente polifarmácia, apoio ao cuidado diário e qualidade do sono estiveram associadas ao grau de sobrecarga do cuidador. Estes achados destacam fatores potencialmente modificáveis e reforçam a importância de abordagem centrada na família de crianças e adolescentes com complexidade médica.

## INTRODUCTION

 Children with Medical Complexity (CMC) are defined as those who often have more than one chronic condition and experience limitations in physical structure and/or function, activity performance, and social participation. These limitations lead to increased utilization of healthcare services, requiring interdisciplinary and intersectoral care.^1^ The prevalence of this condition has been rising globally with the increase in chronic conditions among the pediatric population, which affects approximately 20% of children and adolescents.^1,2^ This increase represents an epidemiological shift due to advancements in technologies that enable survival in numerous critical conditions.^1,3^ However, this survival comes with challenges such as dependence on assistive technologies, polypharmacy, and higher care demands, resulting in significant healthcare costs and profound family impacts.^1^

 The caregiver is the primary individual responsible for providing assistance and supervision to these children and adolescents, taking roles that include routine adjustments, health literacy, specialized care skills, and decision-making.^4^ This dedication can have numerous consequences, particularly due to the high frequency of consultations, hospitalizations, therapies, costs, care fragmentation, and emotional responses to the unfulfilled expectations of an idealized child.^2^ Previous studies have shown that caregivers often sacrifice their lives to provide care, which occupies most of their time, limiting or preventing social involvement and self-care.^5,6^ This context leads to significant caregiver burden. 

 Caregiver burden is defined as the multifaceted stress experienced by a caregiver over time while caring for a family member or loved one.^7^ Its consequences include health deterioration, reduced quality of life, and physical and psychological harm. Additionally, it can compromise the quality of care provided to the ill family member.^4,8^ To mitigate these negative impacts, it is essential to identify the presence of caregiver burden and associated factors to implement strategies for health promotion and harm reduction.^6^

 The impact of chronic illness on family dynamics is well established, particularly in life-threatening conditions. A recent systematic review and meta-analysis found that parents of children with chronic illnesses are substantially more likely to experience mental health challenges, with prevalence rates of 35% for clinical depression and 57% for anxiety, compared to 19 and 38% in the general population, respectively.^9^ Nevertheless, despite the growth of scientific production, substantial gaps in the literature remain, and "support health and well-being of parents, caregivers, and family members" has been identified as one of the priority areas for further investigation.^10^ Most studies address specific chronic conditions, particularly childhood cancer, and fewer studies have been conducted in low- and middle-income countries.^3,4,8^ In these contexts, the burden may be further exacerbated by socio-economic vulnerabilities and limited healthcare infrastructure, making the identification of modifiable risk factors a priority. 

 Thus, considering the rising prevalence of CMC and the importance of caregivers’ physical and emotional health, this study aims to evaluate the factors associated with greater caregiver burden in families of CMC and to provide insights to guide the management of these cases, improving the well-being of children, adolescents, and their families, while addressing a gap in the evidence in resource-limited settings. 

## METHOD

 This is an observational, analytical, cross-sectional study. Its design and reporting followed the STrengthening the Reporting of OBservational studies in Epidemiology (STROBE) guidelines for cross-sectional studies.^11^

 The study was conducted at the General Outpatient Clinic for Children with Chronic Conditions and Special Health Care Needs (Ambulatório Geral de Crianças com Condições Crônicas e Necessidades Especiais de Saúde) of the Institute of Children and Adolescents, Clinics Hospital of the University of São Paulo’s Faculty of Medicine, Brazil. As the largest hospital in Latin America, this facility primarily assists patients in the public healthcare system. It specializes in managing chronic conditions, typically involving multiple diagnoses that result in physical and/or functional limitations frequently requiring intensive care. At this outpatient clinic, care is delivered through a care coordination model led by a general pediatrician. While patients are followed by multiple specialties according to their diagnoses and comorbidities, the general pediatrician integrates specialist recommendations, prioritizes care, and coordinates an individualized, patient- and family-centered care plan.^12^

 The study population comprised primary caregivers of children and adolescents aged 0–18 years diagnosed with chronic physical, behavioral, emotional, or developmental conditions. These patients require higher-intensity, higher-quality care than the general population. All eligible caregivers were invited to participate on the day of the outpatient visit. Patients who attended appointments without their primary caregivers or those residing in institutional settings were excluded from the study. 

 Sample size calculation considered a two-sided significance level of 5% (α=0.05), a β-error of 20%, and the inclusion of 18 predictor variables in the final model. The expected coefficient of determination (R^2^=0.129) was derived from a previous study that evaluated predictors of caregiver burden among mothers of children with chronic conditions, representing the proportion of variance in caregiver burden explained by the set of independent variables included in the model.^13^ Accounting for an additional 10% margin for potential losses or exclusions, the minimum required sample size was estimated at 166 participants. 

 Two previously trained researchers approached caregivers of children and adolescents in the waiting area on the day of the outpatient visit and conducted interviews after written informed consent was obtained. The interviews lasted between 9 and 21 min, with a mean duration of 14 min. 

 Caregiver burden was assessed using the short version of the Zarit Burden Interview, translated into Portuguese and validated for use in Brazil,^14,15^ which measures perceived subjective burden, encompassing emotional, social, and personal impacts associated with the caregiving role. This version includes seven items, categorized on a Likert scale (1–5), that evaluate the time spent on self-care by the caregiver, expressions of stress and distress, impacts of caregiving on friends and family, feelings of exhaustion, perceptions of health deterioration, a sense of losing control of one’s life, and feelings of being overwhelmed by caregiving responsibilities. Previous studies have demonstrated good internal consistency (Cronbach’s alpha=0.83). The sum of the seven items yields a final score ranging from 7 to 35, classifying the burden as mild (up to 14 points), moderate (15–21 points), or severe (above 22 points).^16^ The final score was considered the dependent variable in this study, with all other variables treated as independent: variables related to the children and adolescents’ characteristics (age, gender, and ethnicity), disease severity (associated cerebral palsy, dependency on assistive device, polypharmacy, dependency on daily life activities, frequency of medical or rehabilitation appointments, hospitalizations), caregiver attributes (age, gender, marital status, and education level), and life context (income, support on daily care, religion or spirituality, sleep quality, and home care) were obtained using a semi-structured questionnaire designed by the researchers. 

 Stata 12 (StataCorp LLC, College Station, USA) was used for the analyses. Data were tabulated and subjected to descriptive and inferential analysis. Numerical variables were described using measures of central tendency (mean or median) and dispersion (standard deviation [SD] or interquartile range [IQR]). Categorical variables were described using absolute and relative frequencies. 

 Bivariate analyses were initially performed using the chi-square test to explore associations between caregiver burden and 18 independent variables. In these analyses, caregiver burden was classified into three categories (mild, moderate, and severe). Subsequently, a multivariable Poisson regression model with robust variance estimation was fitted, in which caregiver burden was dichotomized into mild versus moderate/severe, focusing on clinically relevant burden. Variables associated with the outcome at a p<0.20 in the bivariate analyses were considered for inclusion in the multivariable model. Results are presented as prevalence ratios with 95% confidence intervals, and statistical significance was defined as p<0.05. No additional adjustment for confounding factors was applied. 

 The study was approved by the Ethics Committee for Research Project Analysis at the Clinics Hospital of the University of São Paulo’s Faculty of Medicine (CAEE: 70105623.4.0000.0068). Ethical principles outlined in Brazilian Resolution No. 466/12 were followed. As an ethical safeguard, participants identified as having moderate to severe caregiver burden were offered referral for psychological care within the healthcare network. 

## RESULTS

**Figure 1:**
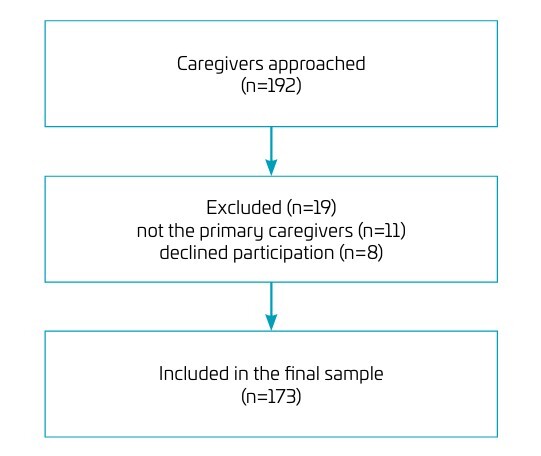
Flowchart of caregiver recruitment, exclusions, and inclusion in the final sample.

 Between November 2023 and October 2024, a total of 173 caregivers were included in the study (Figure 1), the majority of whom were mothers (88.4%). The caregivers were predominantly young to middle-aged adults with relatively low socioeconomic resources, as reflected by limited household income, although most had completed more than 8 years of formal education and lived with a partner. Spirituality emerged as an important coping resource, whereas only about half of the caregivers reported receiving support in daily care. Most of their children were older than 6 years and adolescents with chronic, clinically complex conditions, predominantly cardiac and neurological disorders, often associated with cerebral palsy. Most children and adolescents required ongoing medical management, including polypharmacy, and a substantial proportion depended on assistive devices and support for activities of daily living (Table 1). 

**Table 1:** Demographic and clinical characteristics of the caregiver and the child/adolescent (n=173).

Caregiver		Median (IQR)
Age (years)		39.0 (32.0–45.0)
Per capita income*		44.3 (30.9–70.8)
		n (%)
Gender		
	Female	162 (93.6)
Marital status		
	With partner	110 (63.6)
Educational level		
	More than 8 years of study	124 (71.7)
Receives sufficient support in daily care		94 (54.3)
Relies on religion or spirituality		141 (81.5)
Child/adolescent		Median (IQR)
Age (months)		91.0 (61.0–128.0)
Gender		
	Female	82 (47.4)
Ethnicity†		
	White	98 (56.7)
Category of the main diagnosis		
	Cardiologic	84 (48.6)
	Neurologic	67 (38.7)
	Respiratory	7 (4.1)
	Others	15 (8.6)
Associated cerebral palsy		50 (28.9)
Dependent on assistive devices‡		103 (59.4)
Polypharmacy§		124 (71.7)
Dependent on assistance for activities of daily living		99 (57.2)
Adequate sleep		138 (79.7)
Frequent/prolonged hospitalization in the last 6 months//		18 (10.4)
Weekly medical or rehabilitation appointments		85 (49.1)
Access to home care		13 (7.5)

IQR: interquartile range.

*Per capita income in relation to the percentage of the minimum per capita income in Brazil; †Non-White includes Black, Mixed race, Indigenous, and other self-reported ethnicities; ‡Gastrostomy, tracheostomy, wheelchair, etc.; §Defined as "two or more daily medications," according to Ewig et al.^17^; //Defined by the authors as more than three hospitalizations and/or one hospitalization for more than 1 month.

**Figure 2:**
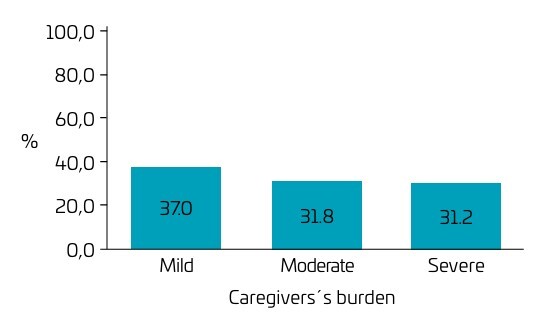
Caregiver burden distribution according to the Zarit Burden Interview.

 Caregiver burden scores on the Zarit scale ranged from 7.0 to 35.0 points (M=17.5, SD=7.4), and the scale demonstrated good internal consistency in our sample (Cronbach’s α=0.866). The mean (SD) Zarit scores for each burden category were 9.8 (2.3) for mild burden, 17.6 (2.0) for moderate burden, and 26.5 (4.1) for severe burden. Figure 2 illustrates the distribution of burden severity across the three categories. 

 The factors associated with a higher prevalence of moderate/severe caregiver burden were female gender, lack of support for daily care, polypharmacy, and the child or adolescent not having adequate sleep. Although not statistically significant, 66.7% of caregivers of infants reported scores consistent with severe burden (Table 2). 

**Table 2:** Association between caregiver and child/adolescent characteristics, clinical factors, and the degree of caregiver burden on bivariate analysis (n=173).

		Mild burden n (%)	Moderate burden n (%)	Severe burden n (%)	p-value*
Caregiver’s age 40 or more years old		33 (41.8)	21 (26.6)	25 (31.6)	0.435
Female caregiver		56 (34.6)	53 (32.7)	53 (32.7)	**0.037**
Caregivers with a partner		37 (33.6)	40 (36.4)	33 (30.0)	0.218
Caregiver’s educational more than 8 years of study		49 (39.5)	38 (30.7)	37 (29.8)	0.549
Per capita income†					
	Q1 (≤25th percentile)	14 (32.6)	12 (27.9)	17 (39.5)	0.526
	Q2 (25th–50th percentile)	16 (35.6)	18 (40.0)	11 (24.4)	
	Q3 (50th–75th percentile)	18 (35.3)	18 (35.3)	15 (29.4)	
	Q4 (≥75th percentile)	15 (45.5)	7 (21.2)	11 (33.3)	
Receives sufficient support in daily care		42 (44.7)	29 (30.8)	23 (24.5)	**0.042**
Reliance on religion or spirituality		50 (35.5)	44 (31.2)	47 (33.3)	0.435
Patient age					
	Infant	1 (11.1)	2 (22.2)	6 (66.7)	0.129
	Preschool-aged	18 (37.5)	20 (41.7)	10 (20.8)	
	School-aged	24 (36.9)	19 (29.2)	22 (33.9)	
	Adolescent	20 (41.7)	12 (25.0)	16 (33.3)	
Female child/adolescent		26 (31.7)	25 (30.5)	31 (37.8)	0.180
White child/adolescent‡		37 (37.8)	32 (32.6)	29 (29.6)	0.870
Associated cerebral palsy		16 (32.0)	20 (40.0)	14 (28.0)	0.333
Dependent on assistive devices§		37 (35.9)	38 (36.9)	28 (27.2)	0.175
Polypharmacy//		39 (31.4)	44 (35.5)	41 (33.1)	**0.050**
Dependency on daily life activities		33 (33.3)	33 (33.3)	33 (33.3)	0.511
Adequate sleep		56 (40.6)	45 (32.6)	37 (26.8)	**0.034**
Frequent/prolonged hospitalization in the last 6 months¶		7 (38.9)	5 (27.8)	6 (33.3)	0.928
Weekly medical or rehabilitation appointments		33 (38.8)	25 (29.4)	27 (31.8)	0.792
Home care		5 (38.4)	4 (30.8)	4 (30.8)	0.993

*χ^2^ test; †Per capita income in relation to the percentage of the minimum per capita income in Brazil; ‡Non-White includes Black, Mixed race, Indigenous, and other self-reported ethnicities; §Gastrostomy, tracheostomy, wheelchair, etc.; //Defined as "two or more daily medications," according to Ewig et al.^17^; ¶Defined by the authors as more than three hospitalizations and/or one hospitalization for more than 1 month.

Bold indicates p-values <0.05.

 In the multivariable Poisson regression model with robust variance estimation, polypharmacy was associated with a higher prevalence of moderate/severe caregiver burden. In contrast, the presence of support for daily care and adequate sleep was associated with a lower prevalence of the outcome. Child age group, child sex, and use of medical devices were not significantly associated with caregiver burden in the multivariable model. Caregiver sex showed a trend toward a higher prevalence of moderate-to-severe burden, although the association did not reach statistical significance (Table 3). 

**Table 3:** Associations between caregiver and child/adolescent characteristics, clinical factors, and the prevalence of moderate/severe caregiver burden estimated by Poisson regression with robust variance (n=173).

	PR	95%CI	p-value*
Child age ≥6 years	0.90	0.72–1.13	0.370
Female child/adolescent	1.11	0.89–1.38	0.357
Female caregiver	2.16	0.90–5.23	0.086
Support in daily care	0.79	0.63–0.99	**0.039**
Use of assistive devices†	0.91	0.72–1.15	0.417
Adequate sleep	0.78	0.63–0.97	**0.027**
Polypharmacy‡	1.48	1.08–2.02	**0.015**

*Multivariable poisson regression model with robust variance estimation; †Gastrostomy, tracheostomy, wheelchair, etc.; ‡Defined as "two or more daily medications," according to Ewig et al.^17^

PR: prevalence ratio; CI: confidence interval.

Bold indicates p-values <0.05.

## DISCUSSION

 Caregivers of patients with chronic conditions face a high risk of burden, characterized by reduced quality of life and physical and psychological harm. These negative impacts are particularly concerning for caregivers of CMC, as the need for care often persists over many years.^6^ In our study, caregiver burden was frequent, with a substantial proportion of caregivers experiencing moderate or severe burden. We identified that caregivers managing polypharmacy for children and adolescents are at greater risk of moderate to severe burden. Conversely, better sleep quality and caregiving support mitigate these negative effects. These findings are significant because they indicate that certain measures can be implemented in the care provided to these patients, improving caregivers’ well-being. 

 Polypharmacy, defined in pediatrics as the simultaneous use of two or more medications, is a common aspect of managing patients with chronic conditions.^17^ This practice poses significant challenges for caregivers, increasing the complexity of care. Medication administration requires careful organization, adherence to schedules, and poses a greater risk of side effects, often causing anxiety over errors, especially among caregivers without formal healthcare training. Moreover, polypharmacy may contribute to increased financial burden and further exacerbate the economic strain associated with caregiving, especially when access to medications through public or institutional coverage is limited. 

 In our study, polypharmacy emerged as the most robust finding, being associated with a higher prevalence of moderate to severe caregiver burden, with caregivers of children exposed to multiple medications showing a 48% higher prevalence compared with those not exposed to polypharmacy. Previous studies have similarly linked polypharmacy to caregiver burden, corroborating our findings.^17-20^ However, research in pediatric populations remains limited. 

 The challenges of polypharmacy are often compounded by fragmented care. CMCs are typically managed by multiple healthcare providers, receiving therapeutic instructions from various sources. This increases the risk of adverse effects, hampers medication adherence, and complicates management for caregivers. Strategies to alleviate caregiver burden related to polypharmacy include implementing coordinated and family-centered care approaches.^21,22^ Building strong relationships between healthcare providers and patients, adopting shared decision-making processes, regularly reviewing treatment plans to eliminate unnecessary prescriptions, and providing caregiver health literacy can reduce the negative impacts of polypharmacy.^23,24^

 Healthcare professionals managing patients with chronic conditions should address all aspects of caregiving, extending beyond symptom control to include factors that influence caregiver quality of life. Sleep quality is one such critical factor that warrants attention. Evidence from the literature indicates that children and adolescents with chronic illnesses show a higher frequency of sleep disorders compared to their healthy peers, which supports the systematic inclusion of sleep assessment in the clinical follow-up of these patients.^25^ This poorer sleep quality is also reported among family members, being associated with anxiety, parental stress, and exacerbated by nighttime caregiving demands.^26^

 Our study’s findings align with prior research suggesting that adequate sleep may play a protective role against caregiver burden, with caregivers reporting that their children slept well showing an estimated 22% lower prevalence of moderate-to-severe burden. From a clinical perspective, sleep quality may be understood as a modifiable factor that influences the daily caregiving experience, rather than merely a secondary symptom of chronic illness. Interventions aimed at improving sleep hygiene and addressing sleep disturbances in both children and caregivers may therefore represent a feasible strategy to mitigate caregiver burden and promote family well-being.^27^

 Caregiving support is another contextual factor in mitigating burden. In our study, the presence of support in daily care was associated with an estimated 21% lower prevalence of moderate-to-severe caregiver burden. indicating that shared caregiving responsibilities, although not eliminating burden entirely, meaningfully reduce the likelihood of more intense overload. Consistent with our findings, numerous studies have highlighted the importance of support systems in mitigating the adverse effects of caring for patients with chronic conditions.^6,28^ This is especially relevant in low- and middle-income countries, where many families cannot afford professional support for the care of children with CMC, who often require intensive attention. Strengthening informal support networks, fostering caregiver networks, and advocating for public policies such as home care programs, access to professional care support, even if temporary during the most challenging periods, and referrals to support groups may improve caregiver outcomes.^6,8,29^

 Gender disparities in caregiving roles were evident in our study, with women comprising most of the caregivers interviewed at the outpatient visits. In the multivariable Poisson regression model, a trend toward an association between caregiver sex and the outcome was observed, with female caregivers presenting a higher prevalence of the outcome compared with male caregivers. Although this association did not reach statistical significance and was characterized by a wide confidence interval, likely reflecting the small proportion of male caregivers in the sample, the direction of the association is consistent with prior literature. This finding is consistent with existing literature showing that women experience higher levels of caregiver burden, emotional stress, and responsibility in the context of chronic illness care.^4,5,30^ Such disparities reflect persistent social norms that disproportionately assign caregiving roles to women, often accompanied by greater self-imposed expectations and limited opportunities for shared caregiving. Addressing this issue requires healthcare professionals to encourage caregivers to seek help and share responsibilities, particularly by involving fathers and strengthening support networks to reduce the burden placed on women. 

 The aspects discussed above demonstrate that quality care, encompassing comprehensiveness, care coordination, and patient- and family-centered care, can potentially reduce caregiver burden. 

 This study is not without limitations. First, the cross-sectional design does not allow for causal inferences, only associations between analyzed factors and caregiver burden. Additionally, the sample was drawn from a single tertiary hospital via convenience sampling, limiting the generalizability of the findings to other populations. The sample was also relatively homogenous, primarily comprising low-income families, which constrained our ability to analyze the social impacts of caregiving. Finally, the absence of data on the duration of the child’s diagnosis and the lack of objective assessments of caregivers’ mental health may have limited a more comprehensive understanding of how disease chronicity and caregiver psychological status interact with caregiver burden. 

 Despite its limitations, this study contributes to the understanding of caregiver burden among families of CMC by identifying factors associated with moderate-to-severe burden. Our findings highlight the relevance of contextual characteristics and care-related needs, particularly polypharmacy, availability of support for daily care, and sleep quality, in shaping the experience of caregiver burden, with gender-related differences in caregiving roles appearing as a secondary and less consistent finding. 

 By focusing on modifiable factors within clinical practice, these results underscore the importance of incorporating a family-centered perspective into the follow-up of children and adolescents with complex chronic conditions. Rather than establishing causal relationships, the observed associations suggest areas that may warrant greater attention during clinical encounters, including caregiving support, sleep-related difficulties, and the complexity of treatment regimens. 

 The findings also reinforce the need for coordinated, comprehensive care approaches that account for both the child’s clinical needs and the caregiving context. Future research, particularly longitudinal and intervention studies, is needed to further examine how changes in these factors over time may influence caregiver burden and to evaluate strategies to support caregivers of children and adolescents with complex chronic conditions. 

## Data Availability

The database that originated the article is available with the corresponding author.
